# Bare Iron Oxide Nanoparticles for Magnetic Harvesting of Microalgae: From Interaction Behavior to Process Realization

**DOI:** 10.3390/nano8050292

**Published:** 2018-05-01

**Authors:** Paula Fraga-García, Peter Kubbutat, Markus Brammen, Sebastian Schwaminger, Sonja Berensmeier

**Affiliations:** Bioseparation Engineering Group, Department of Mechanical Engineering, Technical University of Munich, Boltzmannstr 15, 85748 Garching, Germany; peter.kubbutat@tum.de (P.K.); markus.brammen@outlook.de (M.B.); s.schwaminger@tum.de (S.S.)

**Keywords:** magnetic nanoparticles, bare iron oxides, magnetic separation, microalgae, harvesting, recovery efficiency, electrostatic interaction, adsorption, adhesion, scale-up

## Abstract

Microalgae continue to gain in importance as a bioresource, while their harvesting remains a major challenge at the moment. This study presents findings on microalgae separation using low-cost, easy-to-process bare iron oxide nanoparticles with the additional contribution of the upscaling demonstration of this simple, adhesion-based process. The high affinity of the cell wall for the inorganic surface enables harvesting efficiencies greater than 95% for *Scenedesmus ovalternus* and *Chlorella vulgaris*. Successful separation is possible in a broad range of environmental conditions and primarily depends on the nanoparticle-to-microalgae mass ratio, whereas the effect of pH and ionic strength are less significant when the mass ratio is chosen properly. The weakening of ionic concentration profiles at the interphase due to the successive addition of deionized water leads the microalgae to detach from the nanoparticles. The process works efficiently at the liter scale, enabling complete separation of the microalgae from their medium and the separate recovery of all materials (algae, salts, and nanoparticles). The current lack of profitable harvesting processes for microalgae demands innovative approaches to encourage further development. This application of magnetic nanoparticles is an example of the prospects that nanobiotechnology offers for biomass exploitation.

## 1. Introduction

The steady growth of the human population is driving the search for alternative food and energy sources. In this context, the availability of nonconventional bioresources is continuously being examined and their cultivation processes further developed. However, all the positive and negative issues related to their exploitation must be taken into account. At the same time, the accelerating changes in the modern world are leading to the use of many new materials or simply to taking advantage of new features of existing ones. These developments often do not occur simultaneously with advances in the requisite technologies and processing forms, which nevertheless is a necessity for real progress to take place.

There is a strong industrial interest in microalgae exploitation as a biomaterial source for many applications, especially for nutrition or as biofuel feedstock. Moreover, biorefining—to produce high-value products such as pigments, vitamins, omega-3 fatty acids, recombinant proteins, or antioxidants, to name just a few—is also increasingly becoming interesting for the medicinal, cosmetic, and pharmaceutical industries, among others [1,2,3,4,5,6]. The two main advantages of working with microalgae are: (1) they only need light, water, carbon dioxide, and some salts to grow; and (2) they do not compete with conventional crop cultivation [7]. Much research is currently being conducted on microalgae cultivation, and very promising results have been achieved in increasing the performance of production routes with different approaches [8,9,10,11]. Particularly in open thin-layer cascade photobioreactors, which are more profitable from the economic point of view for large scale cultivation, cell densities as high as 50 g/L have recently been realized [12]. Nevertheless, one bottleneck at the moment is the lack of efficient procedures for separating, concentrating, and dewatering microalgae on a large scale. The small size of the microalgae, their low concentration after cultivation, and the high charge density of their cell walls enable them to remain as stable suspensions and are the reason for the expensive downstream processing, which can account for 20%–60% of total production costs [13,14,15]. At the moment, the conventional methods applied for microalgal biomass harvesting are flocculation, flotation, and sedimentation for the thickening steps and centrifugation and filtration for dewatering [16]. In this work, results of a quite new alternative are presented: the utilization of low-cost, bare iron oxide nanoparticles (BIONs) for separation.

Magnetic nanoparticles in general have been gaining attention for at least the last 20 years, especially due to their magnetic moment. Accordingly, they have been the subject of numerous studies on their interaction with various biomolecules [17,18,19] and with whole cell systems [20,21]. Among others, we are investigating the interactions of magnetic nanoparticles with amino acids [22], peptides [23,24], and proteins [25,26,27], as well as their technical application in high-gradient magnetic separation, for meeting downstream processing goals [28,29]. The main advantages of magnetic separation processes are the high efficiency and the low operating costs, together with simple and fast processing. The idea of using magnetic material to process algae and to apply some form of magnetic separator is not new, probably dating back half a century when separation was incorporated into wastewater treatment [30]. Nevertheless, a strong increase in research on magnetic agents as tools for harvesting microalgae has recently begun, as with new bioeconomy goals, the advantage of using such materials for more efficient concentration and separation routes, potentially in one step, has been recognized. Some works have been published using functionalized magnetic particles [31,32,33,34,35,36]. Of special interest is the direct use of bare iron oxides, which can be easily synthesized and are cost-effective. In 2011, Xu and collaborators published the first article on algae harvesting with bare iron oxide nanoparticles [37]. Later publications highlight the potential of BIONs in bioresource technology [14,38,39,40,41]. Our focus is to combine separation behavior analysis with a demonstration of the feasible application of nonfunctionalized nanoparticles at the liter scale using high-gradient magnetic separation (HGMS). The harvesting efficiency for two different microalgae, *Scenedesmus ovalternus* and *Chlorella vulgaris*, is examined and the detachment step is investigated.

## 2. Materials and Methods

### 2.1. Nanoparticle Synthesis and Characterization

Iron oxide nanoparticle synthesis was carried out using the coprecipitation of Fe^2+^ and Fe^3+^ aqueous salt solutions in an alkaline environment, as previously reported [42]. The atmosphere was not maintained oxygen free. Ferric chloride (FeCl_3_∙6H_2_O) and sodium hydroxide (NaOH) were purchased from AppliChem GmbH, Darmstadt, Germany. Ferrous chloride (FeCl_2_∙4H_2_O) was purchased from Bernd Kraft GmbH, Darmstadt, Germany. Aqueous solutions of ferric chloride (0.2 L, 0.864 kg/L), ferrous chloride (0.2 L, 0.35 kg/L), and sodium hydroxide (2 L, 1.8 mol/L) were prepared with deionized water.

The determination of the morphology and size of the BIONs was carried out using transmission electron microscopy (TEM) (JEM-100CX, JEOL Germany, Freising, Germany). Powder X-ray diffraction (XRD) measurements to examine crystal structure and phase purity of the lyophilized samples were carried out with a Stadi-P diffractometer (STOE & Cie GmbH, Darmstadt, Germany), equipped with a molybdenum source (Ge (111) monochromator, Kα1 radiation (λ = 0.7093 Å)) and a Mythen 1 K detector (DECTRIS Ltd., Baden-Daettwil, Switzerland) in transmission geometry. Data was collected in the range from 2° to 50° (2θ). The software package STOE WinXPOW (STOE & Cie GmbH, Darmstadt, Germany) was used for indexing and refinement purposes. Saturation magnetization measurements were conducted with a superconducting quantum interference device, SQUID (MP MS XL-5, Quantum Design Inc., San Diego, CA, USA) in the range from −628.3 A/m to 628.3 A/m at 300 K. Specific surface areas were calculated from BET-isotherms of nitrogen adsorption (Gemini VII, Micromeritics, Aachen, Germany). The samples were lyophilized before measuring and further dried in the device chamber under vacuum conditions (0.05 mbar).

### 2.2. Microalgae

The experiments were carried out with two algae, *Scenedesmus ovalternus* SAG 52.80 (*S. ovalternus*, see Figure S1a) and *Chlorella vulgaris* Greifswald 9 (*C. vulgaris*, see Figure S1b). Both microalgae species were cultivated and provided by the Institute of Biochemical Engineering of the Technical University of Munich. Cultivation took place in a flat-plate gas-lift photobioreactor in BG-11 medium (1.5 g/L NaNO_3_, 0.04 g/L K_2_HPO_4_, 0.075 g/L MgSO_4_ 7H_2_O, 0.02 g/L Na_2_CO_3_, 0.036 g/L CaCl_2_ 2H_2_O, 0.001 g/L Na-EDTA, 0.006 g/L citric acid, 0.006 g/L ferric ammonium citrate) [43]. Further details on the cultivation of *Scenedesmus ovalternus* SAG 52.80 were recently published by Koller et al. [44]. The interaction studies were performed with cells in the stationary growth phase, after 5–7 days of cultivation. The initial microalgae mass concentration for all the experiments was 0.6 g/L. All chemical agents used were of analytical grade or higher.

### 2.3. Methods and Instrumentation for Interaction Experiments

Gravimetric quantification of BION concentration was determined in 2 mL tubes (Eppendorf, Hamburg, Germany); the samples were dried between 1 and 4 days at 60 °C in an oven (Heraeus UT-6, Thermo Scientific GmbH, Darmstadt, Darmstadt, Germany). For very low BIONs concentrations in the range of 5–50 µg/mL, the determination was carried out spectrophotometrically (Infinite M200 Microplate Reader, Tecan Austria GmbH, Grodig, Austria) as described elsewhere [28]. Mass ratios of BIONs to microalgae were also calculated after gravimetric determination of the dry mass of both. Optical microscope pictures of the algae were taken with an Eclipse 50i microscope, Nikon GmbH, Germany. All laboratory scale experiments were carried out in triplicate.

For all interaction experiments, after shaking, BIONs and algae were incubated for 5 min at room temperature. Then, magnetic separation took place for another 5 min; aliquots of the supernatant were taken and measured spectrophotometrically. Bound and recovered algae were quantified from measurements of the supernatant absorbance at 750 nm using UV–Vis spectrometry. The absorbance of the initial sample before incubation with the BIONs was also measured for all experiments (OD_0_). Harvesting efficiency (or separation efficiency) was calculated according to the following equation:

$$\mathrm{Harvesting}\;\mathrm{efficiency}\;\left[\%\right]=\left(1-\frac{{\mathrm{OD}}_{1}}{{\mathrm{OD}}_{0}}\right)\times 100$$

where OD_1_ corresponds to the absorbance after binding.

For detachment experiments, after binding, the supernatant was carefully removed. The solids were then resuspended in the elution medium, stirred, and separated again using a hand magnet. Recovery efficiency was calculated using the following equation:

$$\mathrm{Recovery}\;\mathrm{efficiency}\;\left[\%\right]=\frac{{\mathrm{OD}}_{2}}{{\mathrm{OD}}_{0}}\times 100$$

where OD_2_ corresponds to the final absorbance of the supernatant.

### 2.4. High-Gradient Magnetic Separation

The separator applied is a prototype for batch processing. A detailed description of a parallel prototype to ours has previously been published [28,45]. The processing device is comprised of a stirred batch adsorption reactor with a peristaltic pump, computer-controlled valves, a bubble detector, and a stirrer. The working volume of the rotor stator magnetic filter is 980 mL. The solenoid has a field strength up to 0.25 T (outer magnet wall), can be automatically switched on or off and is cooled using a water mantle.

All experiments were performed at room temperature. The processing sequence is computer-controlled and runs automatically. The sequence includes the following steps (see [28] for more details):

(1) The adsorption step, which takes place in an external vessel. BIONs and algae are incubated through stirring for 5 min at approximately 200 min^−1^ (RZR 2051, Heidolph Instruments, Schwabach, Germany) and yield a final suspension which is the feedstock for the HGMS process.
(2) The separation step, in which the algae are separated from the broth. While the feedstock flows through the magnetic filter, the electromagnetic field is switched on, enabling the retention of the algae attached to the BIONs inside the filter. At the same time, the medium flows out of the separator.
(3) Wash steps, in which the removal of weakly bound biomaterial and salt takes place. In this phase, a water solution rinses the filter chamber in a closed loop with the rotor on. The magnetic field is still switched on to retain the nanoparticles with the attached biomass, whereas the wash buffer is drained from the separator and collected in an external vessel.
(4) Recovery steps, in which detachment of the microalgae takes place. This is achieved by again pumping water through the filter chamber in a closed loop after resuspending the BIONs with the magnetic field switched off and the stirrer switched on. Afterwards, the magnetic field is switched on again to separate BIONs from the eluted volume. The eluted fractions with the microalgae are collected in an external vessel.
(5) The recycling step, in which the washed BIONs are recovered externally for further application. This step works similar to (4) and leads to a resuspension of the magnetic carrier (magnetic field off and stirrer on).

## 3. Results

### 3.1. Characterization of the Nanoparticles

**Figure 1:**
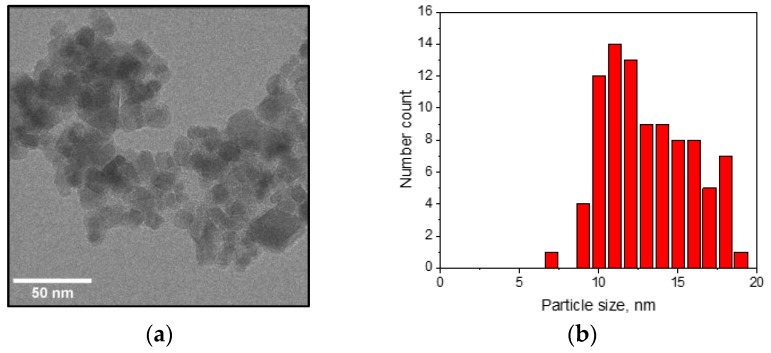

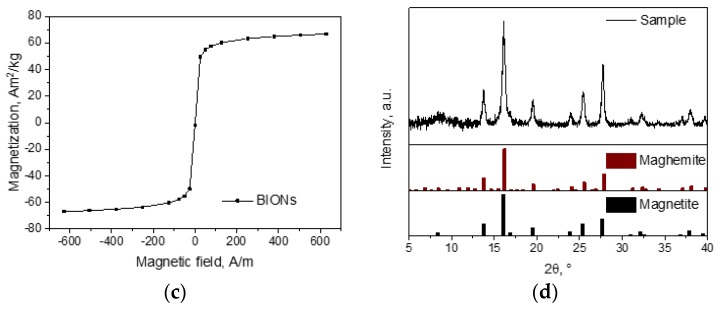
Characterization of the self-synthesized BIONs. (**a**) Transmission electron microscopy image of the nanoparticles; (**b**) particle size distribution histogram from TEM; (**c**) saturation magnetization and (**d**) XRD-spectra (pure maghemite and magnetite crystal phases are added as reference).

The morphology of the BIONs from TEM is presented in Figure 1a. The particle size distribution yields a mean particle diameter of 13.1 ± 2.7 nm (Figure 1b). Saturation magnetization measurements of the nanoparticles resulted in a value of approximately 67 A m^2^/kg (Figure 1c), which indicates a material between the properties of superparamagnetic magnetite (Fe_3_O_4_) and maghemite (γ-Fe_2_O_3_) [46]; a value in this range was expected due to the synthesis conditions in the presence of oxygen. The X-ray diffractometry data in Figure 1d demonstrates that the main crystal form corresponds well with both maghemite and magnetite and, together with the magnetization results (lower than for pure magnetite), leads to the conclusion that maghemite is the dominating crystal structure [46]. The lattice constant a is 8.39 Å with a Scherrer diameter of approximately 9.5 nm. The specific surface area determined from BET-experiments is about 80 m^2^/g.

### 3.2. Interaction of the Microalgae with the BIONs

#### 3.2.1. Adhesion Behavior

**Figure 2:**
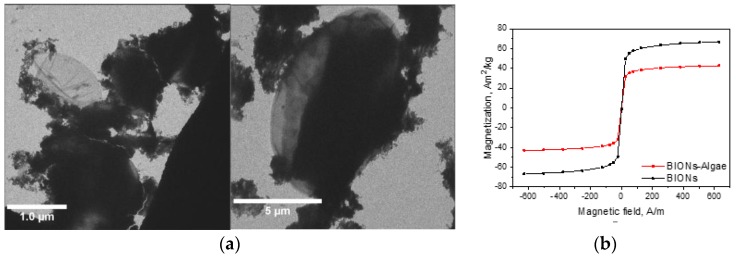
(**a**)Transmission electron microscopy pictures of BIONs and *S. ovalternus* after binding. The BION/microalgae mass ratio was 0.3 g/g at pH 6.75. (**b**) Saturation magnetization data of BIONs after binding to *S. ovalternus* for a BION/microalgae mass ratio of 1.5 g/g. The saturation magnetization of the BIONs before incubation with the microalgae is added as reference.

Attachment experiments were carried out with two algae species, *Scenedesmus ovalternus* and *Chlorella vulgaris,* to test if the findings are more or less representative for more than one microalga. *Scenedesmus* cells are larger than the *Chlorella* cells and appear in small agglomerates (see Supplementary Materials, Figure S1). Transmission electron microscopy pictures of the system after adhesion (Figure 2a) show an agglomerated BION structure which covers part of the microalgae cell wall and is heterogeneously distributed, leaving parts of the cell wall surface free. Saturation magnetizations of the BIONs after incubation with the microalgae help to verify that the material is still superparamagnetic enough after attachment (Figure 2b). The remaining saturation magnetization of approximately 42 A m^2^/kg is above 35 A m^2^/kg, a value which is accepted as a guide limit for microparticle processing in high-gradient magnetic separation [47], and based on our previous experience can also be taken as a reliable orientation for nanoparticle systems. Nevertheless, depending on the magnetic field, the design of the separator, the hydrodynamics during processing, and the density and surface properties of the system, lower saturation can also lead to effective magnetic separation.

**Figure 3:**
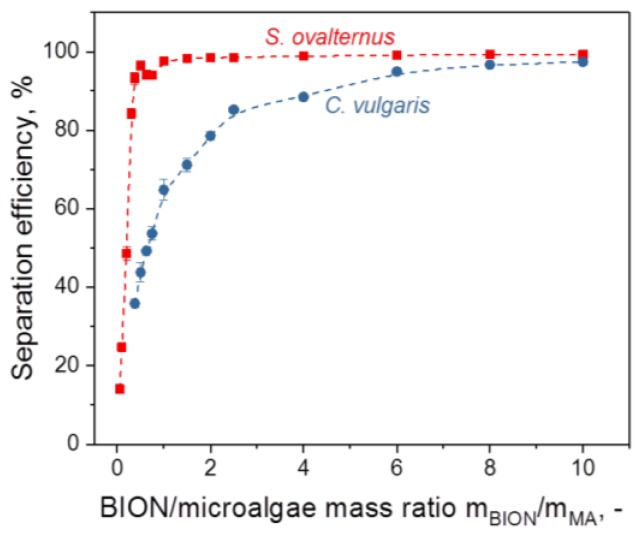
Separation efficiency of *S. ovalternus* und *C. vulgaris* at pH 4 for nanoparticle-to-microalgae mass ratios in the range 0.05–10 g/g.

We wanted to understand the influence of some system parameters on the binding efficiency of microalgae to BIONs and selected nanoparticle dosage (ratio of nanoparticle mass to microalgae dry mass), pH, and salt concentration as the main parameters to be studied, as they have special relevance for most processes in aqueous media and are also important for upscaling. The first task was to determine the optimal mass ratio for separation, meaning the minimum mass of magnetic material per mass of microalgae necessary to achieve the highest separation efficiency. As shown in Figure 3 for pH 4, the separation efficiency depends strongly on the nanoparticle-to-microalgae mass ratio for lower nanoparticle masses; this value is also a function of the microalgae chosen. The most important result is probably the discovery that efficiencies of 95% are achievable for both algae species. Similar results have previously been published for different, mainly coated magnetic particles and several algae species [31,32,36,37,41,48,49,50,51,52].

**Figure 4:**
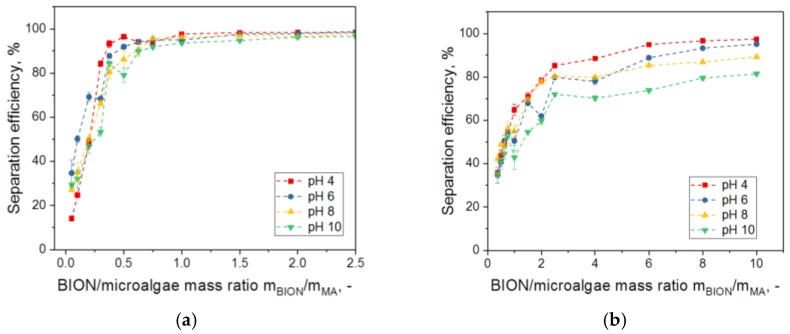
Separation efficiency of *S. ovalternus* (**a**) and *C. vulgaris* (**b**) at different pHs in the relevant nanoparticle dosage range for each.

The separation efficiency shows a very slight dependency on the pH in the relevant mass ratio window; this dependency seems to be more significant for *C. vulgaris* (Figure 4b) than for *S. ovalternus* (Figure 4a). This difference is probably because the mass ratios chosen are too low to achieve saturation values at all pHs. However, in both cases, very high efficiencies are reached for higher BION concentrations. To take a closer look at the pH effect, the recovery efficiency is plotted in relation to the pH for the maximum nanoparticle-to-algae mass ratios in Figure S2. Here, the mass ratio 0.5 g/g was selected as maximum for *S. ovalternus* and 10 g/g for *C. vulgaris*. The efficiency decrease for higher pHs is difficult to recognize for *S. ovalternus* (see again Figure 4a).

Although our results reveal no strong pH effect, previous studies have noted a decreasing tendency for other systems [32,35,36,37,41], which has been connected to the zeta potential of the magnetic nanoparticles. As we have previously reported, the BIONs have an isoelectric point (IEP) close to a neutral pH or higher [23,46], whereas most of the microalgae have a negative zeta potential, at least above pH 4 [13,32,36,40,53,54]. Hence, for pH values below the IEP, the shear plane of the nanoparticles is primarily positively charged, thus facilitating a higher interaction propensity with the primarily negatively charged algae cell wall. Nevertheless, it does not explain the interaction at higher pHs, where both the nanoparticles and algae are negatively charged.

**Figure 5:**
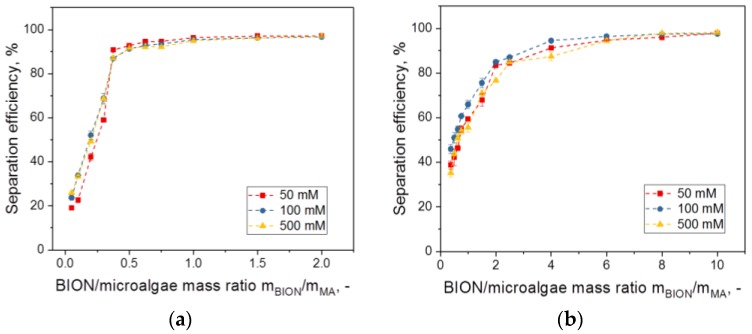
Separation efficiency of *S. ovalternus* (**a**) and *C. vulgaris* (**b**) at pH 4 and for different NaCl concentrations in the respectively relevant nanoparticle-to-microalgae mass ratio.

Experiments with different NaCl concentrations added do not indicate a dependency of the separation efficiency on the salt concentration in the range from 50 to 500 mM, whereas they confirm some dependency upon the pH (Figure 5 and Supplementary Material, Figures S3–S5). The fact that at high ionic strengths (e.g., 500 mM, see Figure S3) no changes in the development of the separation efficiency of the BION-to-microalgae mass ratio are observed could suggest that the shielding of electrostatic forces due to the higher salt concentration at the interface does not have any observable effect. Indeed, the electrostatic interaction may not play a leading role for the interaction. We expected more differences on that point due to changes in the agglomeration behavior of the system.

Generally, *S. ovalternus* is better suited than *C. vulgaris* concerning the amount of BIONs needed for maximal separation efficiency. Therefore, further experiments were conducted only with this microalga. Temperature was tested to assess its impact on the interaction and the results, shown in Figure S6, reveal that for lower temperatures (13 °C), the separation efficiency may be approximately 25% lower than for higher temperatures (40 °C). This positive effect of higher temperatures on separation efficiency has been reported in the literature for *N. maritima* [55] and should therefore be taken into consideration for processing. Nevertheless, the presence of a sufficient mass of magnetic material leads to the highest efficiency for the three temperatures chosen.

#### 3.2.2. Microalgae Recovery

Depending on the final goal of the algae harvesting, their recovery from the magnetic material is an essential task, aside from the separation of the algae from their initial environment using magnetic forces. Recovery experiments have two aims: to obtain the target material (the microalgae) in a pure form and to recover the magnetic carrier for further use (recycling). The first goal is especially important for nutritional or pharmaceutical applications, whereas the second is relevant for lower value products (price per mass unit), e.g., for biofuel use or wastewater treatment, where the main objective of recovering the magnetic material is to enhance the economic feasibility of the whole processing chain.

**Figure 6:**
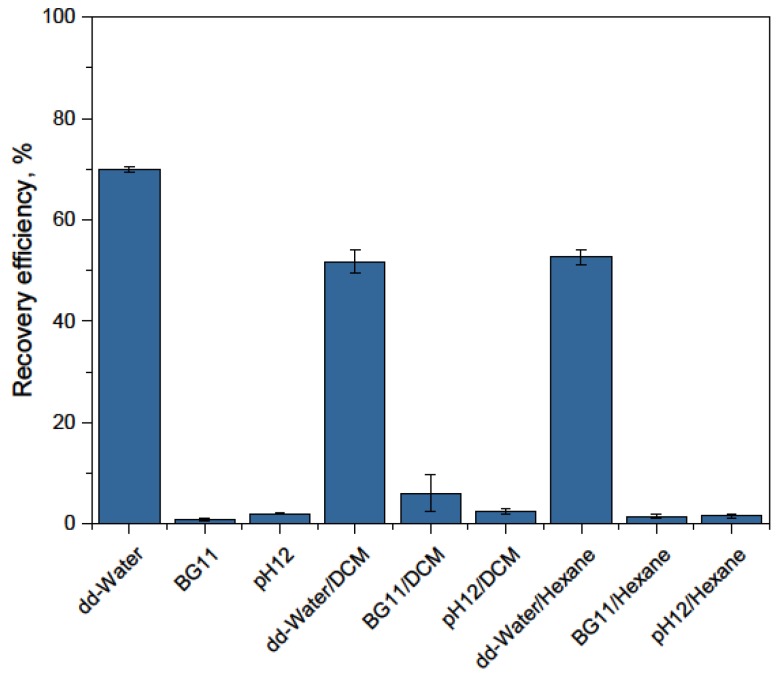
Recovery efficiency of *S. ovalternus* for mass ratio BION/microalgae of 1.5 g/g at pH 8.35 (or pH 12 when directly stated).

The recovery experiments were carried out to gain some indications on the following changes: (1) the dilution effect resulting from the addition of deionized water; (2) the same dilution effect, but in the presence of salt (BG-11 medium); (3) a strong pH shift to the range of lower efficiencies (pH 12); and (4) two phase systems, where changes in the cell wall and a weakening of all ionic and electrostatic effects could lead to recovery enhancement. The results are displayed in Figure 6.

Whereas the addition of an algae-free BG-11 medium or a pH shift did not lead to an observable recovery, all systems with deionized water led to detachment of microalgae. To be certain that desorption merely in the presence of water was not due to cell lysis, the samples were analyzed with optical microscopy. The illustrations of the microalgae after the recovery experiments with deionized water demonstrate that complete processing (magnetic separation first and recovery afterwards) did not alter their morphology (see Figure S7). No higher degree of algae disruption was observed in the pictures, neither before nor after processing. 

The recovery of the microalgae as well as the BIONs is essential for the sustainability and for the economics of the whole processing chain. Nevertheless, recovery has rarely been treated in the literature. Some groups only mention that it would be nice to have [32], while others use strong acids to dissolve the oxides and obtain the algae free of magnetic material [37,40]. Only Prochazkova et al. analyzed the detachment question in depth [36,40], whereas Lee et al. demonstrated the reusability of the particles in several cycles [56]. In our study, the particles are also completely recycled and can immediately be reused for further harvesting purposes. In addition, the algae are recovered in their original state.

### 3.3. Upscaled Processing Using High-Gradient Magnetic Separation

The second goal of this research was to test the transferability of the knowledge gained on the interaction behavior between the nanoparticles and the microalgae to the liter scale. The experiments were run in a high-gradient magnetic separator. The working procedure has been described elsewhere [28]. From preliminary experiments to test the processing parameters with bare iron oxide nanoparticles, a flow velocity of 250 mL/min (approximately 30% pumping performance) was identified as adequate for complete retention of the BIONs during the separation step. For this flow rate, no magnetic material was found in the collected fractions, as verified by spectrophotometric and gravimetric methods. The pilot-plant experiments were only performed with *S. ovalternus*. Quantification of the separation and recovery of algae, salts, and nanoparticles was carried out using spectrophotometric analysis combined with dry-matter determination as well as qualitatively evaluated using visual controls.

The first experiments were carried out at pH 4, which had yielded the best results at the laboratory scale. Experiments with low BION-to-microalgae mass ratio (0.3 g/g) showed lower separation efficiency in the HGMS than that for the same mass ratio at the laboratory scale. Some microalgae were lost during the separation step, while even more mass was lost after the addition of water during the washing step. These effects might be caused by BION loss due to interaction with the tubing as well as loss inside the separator chamber (accumulation of magnetic material in dead zones), which would lower the amount of BIONs available for binding algae. For higher BION mass ratios (e.g., 1 g/g), the separation was entirely successful, but no microalgae recovery with high water volumes was achieved.

Figure S8 shows fractions of the different steps during a harvesting and recovery process using the optimal conditions from the laboratory experiments, i.e., at pH 4 and for 0.5 g_BION_/g_microalgae_. The BIONs were completely retained by the magnetic forces inside the separation chamber and no loss of microalgae was observed during separation (fraction 01 in Figure S8), nor during washing with 3.25 L water (fractions 02 and 03). Through the addition of higher amounts of water and circulation with stirring, the detachment of the microalgae without BION contamination was achieved and can be observed in the green color of the collected fractions (04 to 09 in Figure S8). Each fraction corresponds to the addition of 1 L water; only for the last fraction were 2 L added to wash out the rest of the microalgae. In the end, recovery of the magnetic nanoparticles takes place. Here, again, 4 L water circulate through the system with the magnet off and the rotor on to achieve the complete release of the BIONs from the separator chamber. The separation process works efficiently—salts are recovered primarily during separation and further during the washing step, whereas algae are collected during the recovery steps free of salt and of BIONs. Finally, the BIONs can also be released from the system and used for further processing.

**Figure 7:**
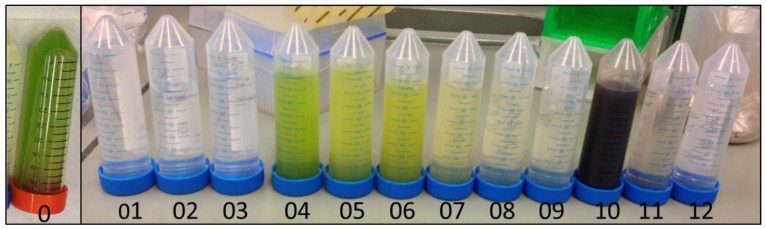
Fractions of the HGMS process with 5 L microalgae suspension, 0.5 g_BION_/g_microalgae_, pH 6.75. Fraction 0 shows the native algae suspension (initial situation); fraction 01 corresponds to the separation step and includes the BG-11 medium; fraction 02 and 03 were collected after washing and are mainly residual salts in water; fractions 04 to 09 were collected during the microalgae recovery steps and contain only microalgae and water; fractions 10 to 12 were collected during the BIONs resuspension step and correspond to the recovery of the nanoparticles.

The next step was then to verify the separation directly in the algae medium conditions after production, thus without pH adjustment or salt addition and at the same time to carry out the process with a higher initial volume. The aim here was to monitor viability under conditions more interesting for industrial harvesting. The initial volume was 5 L of a mixed suspension containing 0.5 g_BION_/g_microalgae_ at pH 6.75. The qualitative demonstration of the progress of the separation procedure is shown in Figure 7, which presents the collected fractions. The initial algae suspension is represented in fraction 0. The next fraction (fraction 01) corresponds to the separation step, where the 5.03 L BION-microalgae mixture flows through the separation chamber with the magnet switched on and without stirring. At the outlet, only the medium is collected. No absorbance can be detected, meaning that neither algae nor BIONs are lost, which is very important for the process and works flawlessly.

After separation, washing eliminates remaining salts which might still be attached to the cell wall, the BIONs, or retained somewhere else in the system. First 2 L water were added and then a further 0.25 L just to wash out the volume captured in the separator bottom. The rotor was switched off and the magnetic field on.

The next steps lead to the recovery of the algae. Each step includes the addition of 1.5 L water which circulates through the system. The rotor is on and the magnetic field off, with the circulation in loop and the additional rotation enhancing the detachment performance of the microalgae. The water loading needs approximately 6 min (250 mL/min); the additional circulation in loop takes place for another 2 min. Afterwards, the rotor is stopped and the magnetic field switched on to retain the BIONs in the separation matrix while the detaching microalgae are pumped out of the device (fractions 04 to 09). The same sequence in repeated for each recovery step.

The final steps lead to the release of the BIONs and their recovery (fractions 10 to 12). In the first two steps, again 1.5 L water are pumped through the system and the stirrer is on, whereas the magnetic field is off. In the last step 4 L water are added to wash out all material residues from the device and the dead zones. Most of the BIONs are found in the first step, but larger agglomerates also appear in the last step.

**Figure 8:**
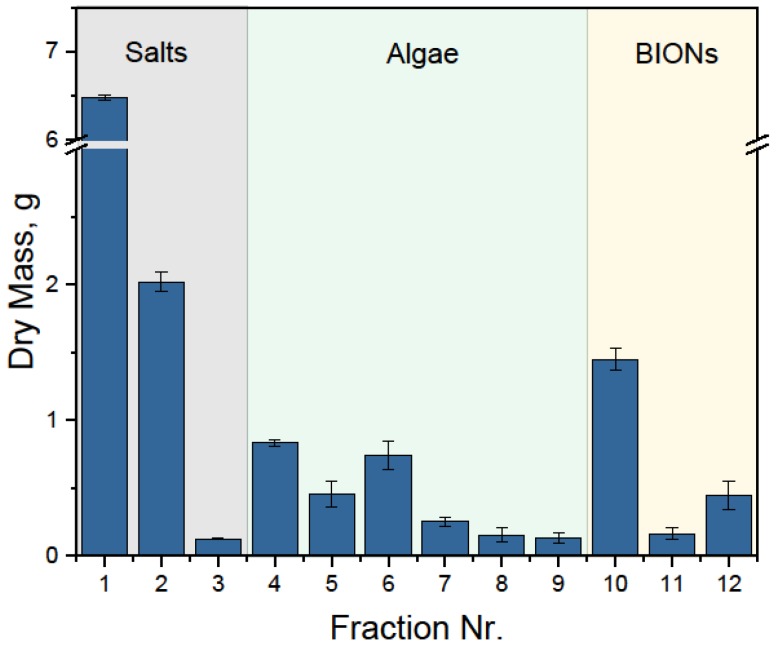
Dry mass of the fractions of the HGMS process with 5 L microalgae suspension, 0.5 g_BION_/g_microalgae_, pH 6.75. The first fraction corresponds to the separation step, where the medium free from BIONs and algae is collected. The other salt fractions correspond to the washing steps. The algae fractions correspond to the recovery steps, in each of which 1.5 L water is pumped through the system and circulated by stirring. The last fractions correspond to the BIONs release from the system.

The algae dry masses in each fraction of Figure 7 are presented in Figure 8 and Table 1. Additionally, Figure S9 shows the fraction mass in dependence of the processing volume. The first feature which can be quickly recognized is that salts and nanoparticles are recovered effectively without requiring high water volumes, whereas the recovery of the algae takes place only through the subsequent washing from the nanoparticles’ surface. The second important point is that some algae probably remain on the nanoparticles’ surface; however, quantifying them is not possible with the methods employed (gravimetric and spectrophotometric) in the presence of the nanoparticles. Furthermore, the experimental procedure leads to some errors in quantification due to the measurement of small sample volumes. At the beginning, the total masses were approximately 3 g microalgae, 1.5 g BIONs, and 8.4 g salts from the BG-11 medium. The salt mass is found in the first fractions with high accuracy. The mass of BIONs detected is higher than the initial value, while the algae mass from the algae fractions is lower than initially. The most plausible explanation for this phenomenon is that 15% of the algae were still attached to the nanoparticles. The relatively slight decrease of algae mass for each washing step leads to the conclusion that further washing would be necessary for complete microalgae recovery. For a more reliable quantification and a proper mass balance, further analytics are necessary, especially at the end of the processing sequence, e.g., thermogravimetric methods accompanied by mass-spectrometry.

These results demonstrate that BIONs enable the complete separation of the algae from the medium where they were initially, and that no mass losses take place, neither of biomass nor of salts, nor of nanoparticles. To the best of our knowledge, no harvesting and recovery process driven in a similar way to that presented here has been published in the literature.

## 4. Discussion

The first goal of this study was to gain a better understanding of the interaction behavior of microalgae with BIONs. By simultaneously considering two microalgae species, both of them interesting in regard to industrial exploitation, it should be possible to observe if the behavior is similar for different species. After comparing our results with others published for bare iron oxides and other species [37,40,41,52], the following can be identified:

- For values above a threshold (saturation value) of the mass ratio of the nanoparticles to the microalgae, the separation efficiency of the microalgae comes close to 100%. The slope and saturation values of the separation efficiency diagram are specific for each system and depend on surface composition, dimensions, and the morphology of the microalgae species as well as of the nanoparticles and the content of the aqueous phase.
- The separation efficiency decreases with increasing pH. However, with a sufficient mass of magnetic material, separation is possible in a very broad pH window. The results of Xu et al. with *Chlorella ellipsoidea* showed a parabolic behavior of the efficiency with the pH with the highest value at neutral pH, but these results represent an exception [37].
- Although in our experiments no dependence was recognizable for NaCl concentrations between 0.05 and 0.5 M, this is probably not a general rule for all salts but depends on the salt and the concentration range. That the impact of ionic strength on separation depends on the salt selected was demonstrated by Procházková, who showed that phosphates have a strong effect and lead to a decreasing separation efficiency, whereas other anions did not [40]. We have also illustrated the high affinity of phosphate or citrate ions for the BION surface in previous work on adsorption of peptides and proteins [57], which explains the lower adsorption of other substances to the surface. Further adhesion experiments should deepen these findings.
- The main parameter is the minimum mass of magnetic material necessary to achieve maximal separation efficiency. Below this value, parameters such as the pH have a strong impact on the separation efficiency, whereas close to this value the effect becomes smaller.

One question of special relevance here is what the main forces responsible for the adhesion process are. We initially considered electrostatic interactions as the leading forces, but the small effects due to pH and salt variation on the separation efficiency are not easy to interpret within the framework of an electrostatically driven adhesion. Regarding this, some points should be highlighted:

- Microalgae outer cell walls have many different functional groups, which are mainly negative, but positive groups are also present [54]. In experiments using bacteria cells, and based on zeta-potential results, Li et al. maintained that nano-size effects together with hydrophobic interactions, rather than electrostatic forces, are responsible for the interaction of the bacteria with the oleate-coated iron oxide nanoparticles used [20]. Cerff and colleagues questioned this outcome, adding that the possible existence of locally-distributed positive charges of, e.g., protonated amino groups, may still make the electrostatic interaction responsible for adsorption [32]. Then, positively charged groups may meet negatively charged groups across the whole pH range, due to the complexity of the components present and the various dissociation constants (the so-called Lewis acid-base properties). Therefore, even when repulsion is expected from the zeta potential values (netto charge) of nanoparticles and algae, the patchwork model for charge distribution on the surface makes interaction possible, as recently presented by us [57]. Furthermore, the zeta potential of the particles depends strongly on the environmental conditions [40,57]. Note that the zeta potential measurement, although a fast, easy, and widely used method for gaining surface charge information, is not completely appropriate for microalgae systems due to the complex medium present, as explained by Lim et al. [31].
- On the other hand, if the results of detachment experiments are taken into account, it is difficult to understand why only the addition of deionized water has such a strong impact on detachment, whereas dilution by adding BG-11 medium and strong pH shift to pH 12 have no impact. One possible explanation is that in both cases, enough salt is still present (in the second case due to the counterions for the pH adjustment) and the ions at the interphase lead to chelate complexation and molecular bridging with the complex cell wall (polysaccharides, lipids, proteins) hampering detachment [58]. In contrast, the addition of salt-free water strongly decreases the ion concentration in the double layer and therefore leads to the cancellation of electrostatic and/or ionic effects and coordination bonding. Procházková et al. obtained low detachment values and used the extended Derjaguin–Landau–Verwey–Overbeek theory to explain this result and to analyze the adhesion mechanism, concluding that this theory is not sufficient to describe the observations and, therefore, covalent forces are probably also involved in the interaction [36]. They worked with coated particles, which could open the door to covalent interactions. In the case of bare iron oxides, as presented in this paper, the presence of covalent forces is difficult to imagine, due to the high efficiency of detachment using salt-free water. Nevertheless, osmotic properties of the algae may be the driving force or at least an important factor. This is an essential question which should be investigated in the future.
- Moreover, interactions in complex systems are always an interplay of several forces. Hydrogen bonds, van der Waals forces, and steric effects can also be expected to play an important role in the interaction and are present across the whole pH range [41,56].
- Finally, the agglomeration behavior of BIONs and of microalgae is influenced by environmental conditions and also impacts harvesting efficiency. Changes in the agglomeration behavior of the system have not yet been considered in studies published on magnetic separation of microalgae, although they are probably one key for better understanding the system dependencies.

The second part of this paper deals with technical-scale processing. The first very relevant circumstance here is that the separation also works successfully at the liter scale, i.e., (1) BIONs are completely retained during separation, (2) algae are almost completely separated from the original medium, and (3) all materials which enter the process—meaning the salts of the medium, the algae, and the BIONs—are recovered at the end with a high degree of purity. Accordingly, BIONs and the use of magnetic forces for separation are perfectly suitable for microalgae harvesting. The agglomeration behavior of the BION/microalgae system has a positive effect with regard to binding and processing. It remains unclear if the effect of the shear forces due to circulation and rotation facilitates detachment, but with enough water, microalgae recovery is possible with very high yields. From the technical point of view, to the best of our knowledge, the only separation processes published with bare iron oxides prior to this study are those of Toh et al. [59] and Hu et al. [50]. By means of automatic high-gradient magnetic separation of 5 L of microalgae, we demonstrate that HGMS can be seen as an appropriate separation technology, which has the advantages of simplicity, low-cost operation, robustness, as well as short processing time, all of which are decisive aspects for microalgae harvesting. Nevertheless, the liter scale experiments only represent a proof of concept. Our goal here is to show that a working process can be driven. We are indeed aware that the greatest challenge for the industrial application of HGMS is to enhance concentration factors reducing the recovery volume employed. In the future, more appropriate detachment methods should be tested. Furthermore, the separator used in this study is still a prototype and requires further improvement. Ongoing investigations are focusing on the optimization of the separating device (geometry, feeding, etc.) and the tubing [60].

## 5. Conclusions

The long-term goal in microalgae processing has to be the development of integrated, low-cost approaches to increase profitability. Through process intensification towards better overall performance in the exploitation of algal biomass, taking advantage of the heterogeneity of these natural resources to obtain multiple products would be achievable [61]. Here, we think that using iron oxide nanoparticles has the potential to open doors. Magnetic harvesting enables the processing of microalgae without any loss of material. Microalgae can be completely exploited, whereas magnetic particles and medium [55] are recycled, which is very important for process economics and sustainability. In addition, the immobilization of algal material on the surface of magnetic nanoparticles is of interest for other application fields, such as wastewater treatment [62,63], medicinal, or pharmaceutical purposes, and even for enhancement of product formation [64]. Another interesting future prospect is combining cultivation and harvesting in in situ separations, as already reported for some systems [65,66,67]. We tested the growth of yeast cells in the presence of our BIONs and observed no recognizable influence on the growth curve of *Saccharomyces cerevisiae* (see Supplementary Materials, Figure S10).

Finally, we want to encourage further work on the development of devices for magnetic-based biomass recovery. This study demonstrates that magnetic separation is a sustainable alternative to processes currently used, but at the moment no separators appropriate for microalgae harvesting exist. Hence, innovation in magnetic plant design and engineering, in particular, is needed to generate new concepts and solutions for industrial algae harvesting in the future.

## Figures and Tables

**Table 1:** Dry mass of the fractions collected as quantified by gravimetry and spectrophotometrically. In the fractions 1–3, no absorbance was detected and therefore their mass should correspond only to the salts. In fractions 4–9, no magnetic material was observed in the samples or collection vessels. Fractions 10–12 mainly contain BIONs, but due to limitations of the quantification methods, identifying the mass of algae in these fractions was not possible.

Process Step	Fraction	Mass, g	Mass_algae_, g	Mass_salts_, g	Mass_BIONs_, g
**Adsorption**	Initial	12.90	2.99	8.42	1.49
**Separation**	1	6.48	0	8.6	0
**Wash Salts recovery**	2	2			
	3	0.12			
**Desorption Algae recovery**	4	0.83	2.55	0	0
	5	0.45			
	6	0.74			
	7	0.25			
	8	0.15			
	9	0.13			
**Recycling BIONs recovery**	10	1.45	*x*	0	2.05 − *x*
	11	0.16			
	12	0.44			
	Final	13.2			

## Supplementary Materials

The following are available online at http://www.mdpi.com/2079-4991/8/5/292/s1, Figure S1: Light microscopy pictures of the microalgae, Figure S2: Separation efficiency in the pH range of 4 to 10, Figure S3: Separation efficiency at different pHs and 500 mM NaCl, Figure S4: Separation efficiency at different pHs and 100 mM NaCl, Figure S5: Separation efficiency at different pHs and 50 mM NaCl, Figure S6: Temperature influence on the separation efficiency of *S. ovalternus*, Figure S7: TEM picture of S. ovalternus after recovery from the BIONs using deionized water, Figure S8: Fractions of the HGMS process with 1 L microalgae suspension, 0.5 g_BION_/g_microalgae_, pH 4, Figure S9: Dry mass of the fractions in dependence of the process volume for the HGMS process with 5 L microalgae suspension, 0.5 g_BION_/g_microalgae_, pH 6.75, Figure S10: In-situ cultivation experiments with 85 mL *Saccharomyces cerevisiae* in YPD-Medium.
