# Supplementary material for: Bare Iron Oxide Nanoparticles for Magnetic Harvesting of Microalgae: From Interaction Behavior to Process Realization
Source: Nanomaterials (Basel). 2018 May 1;8(5):292. doi: 10.3390/nano8050292 (PMC5977306; doi:10.3390/nano8050292)
Supplement: Supplementary file 1 [file nanomaterials-08-00292-s001.pdf]

Supplementary Material

Bare iron oxide nanoparticles for magnetic harvesting of microalgae: from interaction behavior to process realization

P. Fraga-García*†, P. Kubbutat†, M. Brammen, S. Schwaminger, S. Berensmeier*

Bioseparation Engineering Group, Department of Mechanical Engineering, Technical University of Munich, Boltzmannstr. 15, 85748 Garching, Germany; p.fraga@tum.de (P.F.-G.); peter.kubbutat@tum.de (P.K.); Markus.Brammen@outlook.de (M.B.); s.schwaminger@tum.de (S.S.); s.berensmeier@tum.de (S.B.)

* Correspondence: p.fraga@tum.de (P.F.-G.); s.berensmeier@tum.de (S.B.); Tel.: +49-89-28915759 (P.F.-G.); Tel.:+49-89-15750 (S.B.)

† These authors contributed equally to this work.

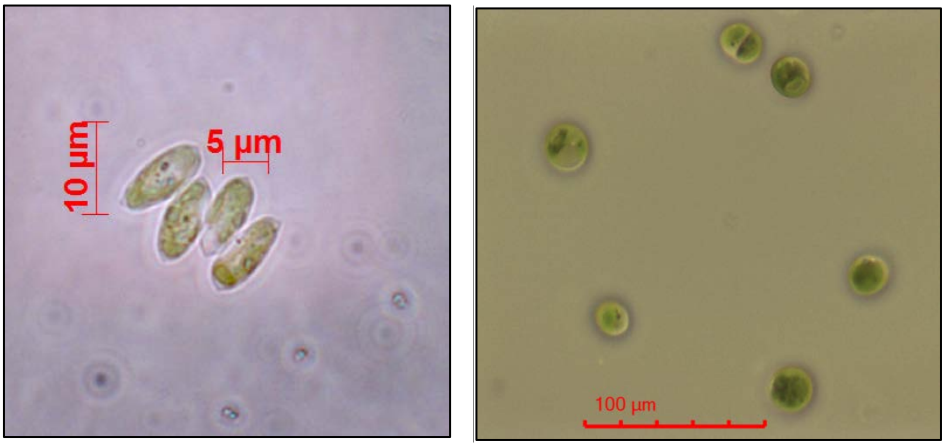

Figure S1: Light microscopy pictures of the microalgae: *Scenedesmus ovalternus* SAG 52.80 (left) and *Chlorella vulgaris* Greifswald 9 (right).

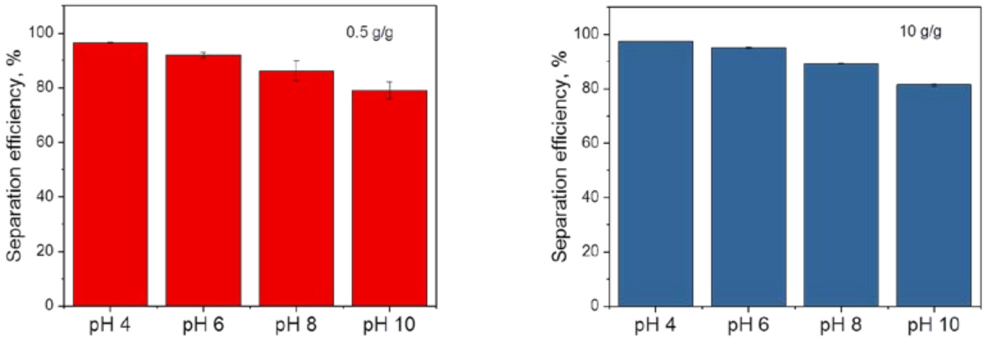

Figure S2: Separation efficiency of *S. ovalternus* (left) and *C. vulgaris* (right) with BIONs in the pH range of 4 to 10 for BION/microalgae mass ratios of 0.5 g/g and 10 g/g respectively.

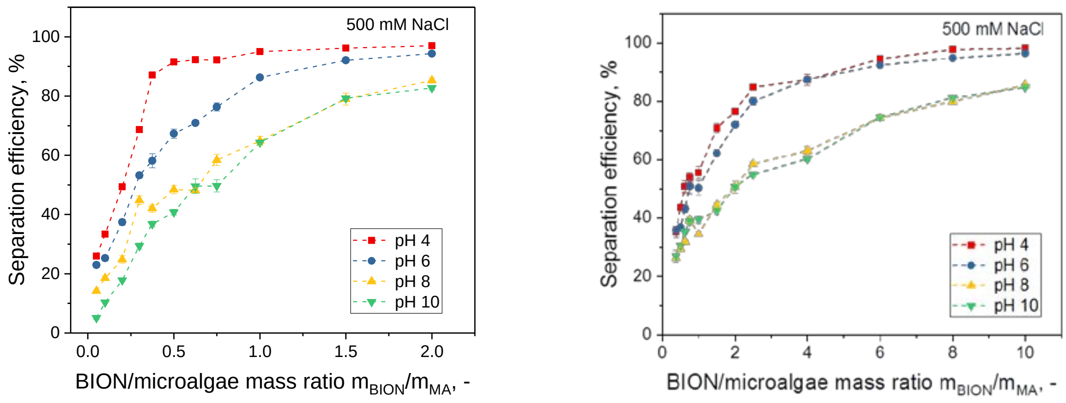

Figure S3: Separation efficiency of *S. ovalternus* (left) and *C. vulgaris* (right) at different pHs and 500 mM NaCl in the respectively relevant nanoparticle dosage range.

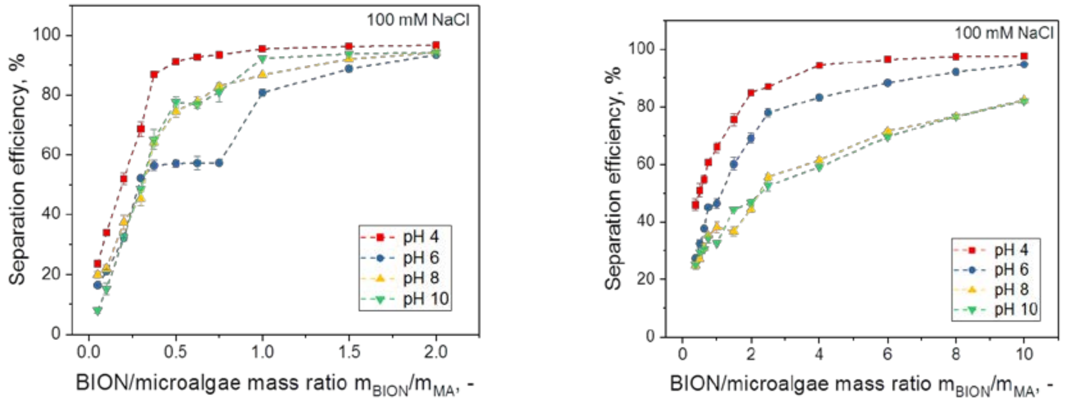

Figure S4: Separation efficiency of *S. ovalternus* (left) and *C. vulgaris* (right) at different pHs and 100 mM NaCl in the respectively relevant nanoparticle dosage range.

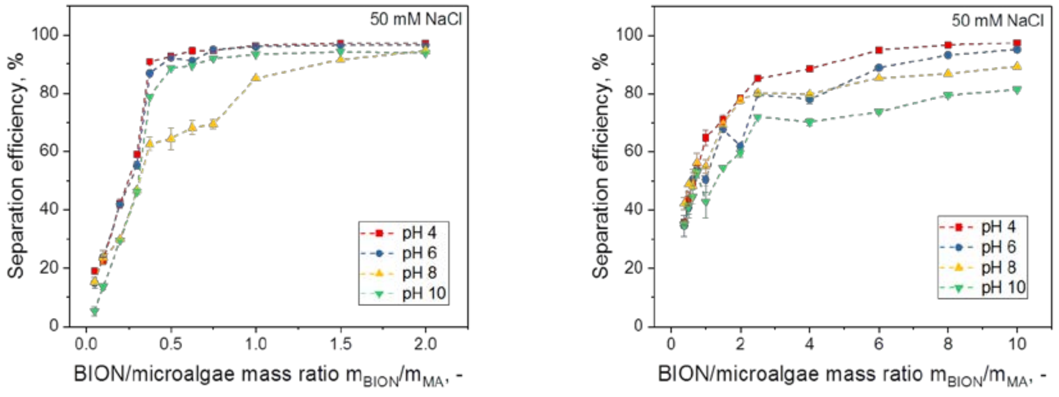

Figure S5: Separation efficiency of *S. ovalternus* (left) and *C. vulgaris* (right) at different pHs and 50 mM NaCl in the respectively relevant nanoparticle dosage range.

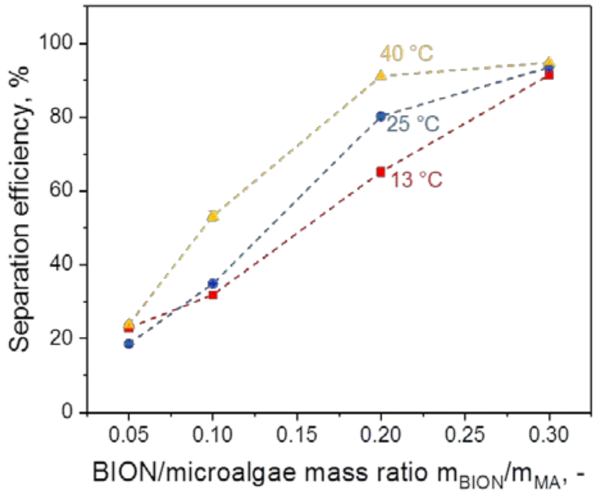

Figure S6: Temperature influence on the separation efficiency of *S. ovalternus* for nanoparticle to microalgae mass ratios between 0.05 – 0.30 g/g at pH 4 and temperatures of 13, 26 and 40°C.

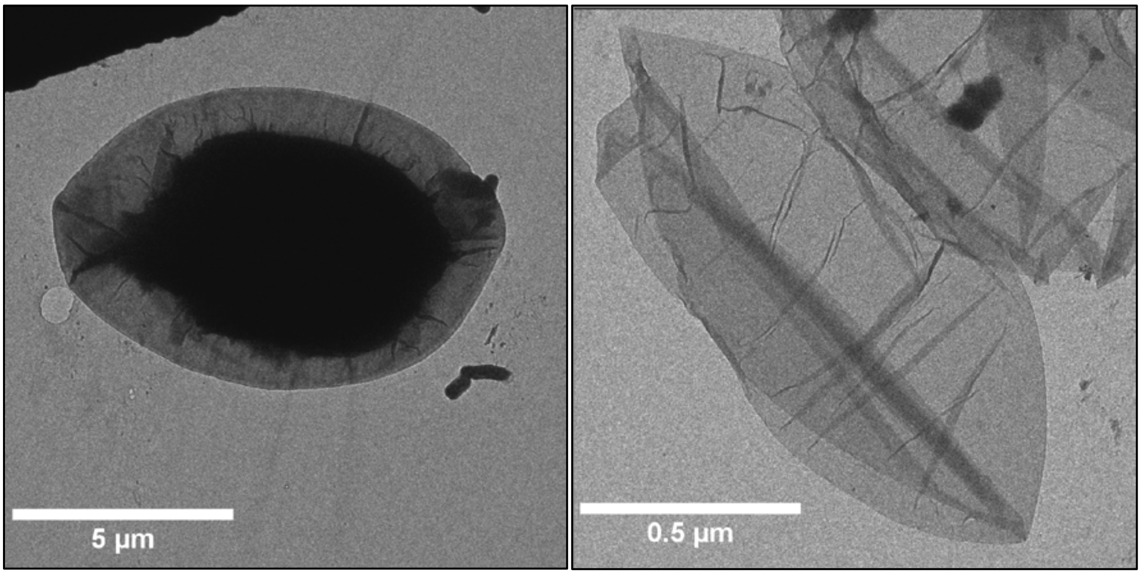

Figure S7: TEM picture of *S. ovalternus* after recovery from the BIONs using deionized water.

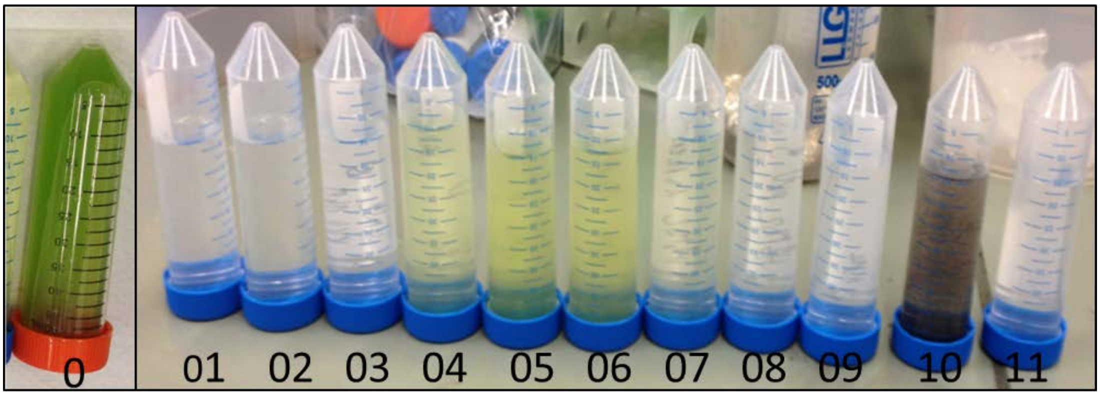

Figure S8: Fractions of the HGMS process with 1 L microalgae suspension, 0.5 $g_{BION}/g_{microalgae}$, pH 4. Fraction 0 shows the native algae suspension (initial situation); fraction 01 corresponds to the separation step and includes mainly BG-11 medium and sodium and chloride ions from the pH adjustment; fraction 02 and 03 were collected after washing and are mainly washed salts in water; fractions 04 to 09 were collected during the microalgae recovery steps and include only microalgae and water; fractions 10 and 11 were collected during the BIONs resuspension step and correspond to the recovery of the nanoparticles.

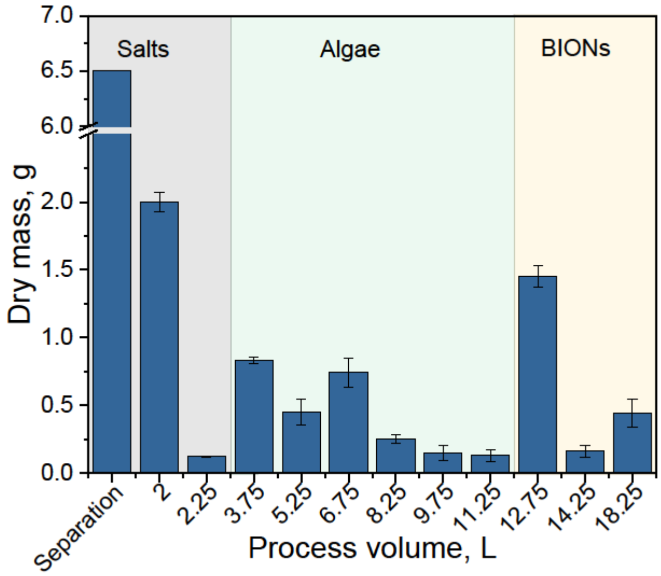

Figure S9: Dry mass of the fractions in dependence of the process volume for the HGMS process with 5 L microalgae suspension, 0.5 gBION/gmicroalgae, pH 6.75. The first fraction corresponds to the separation step, where the medium free from BIONs and algae is collected. The other salt fractions correspond to the washing steps. The algae fractions correspond to the recovery steps. The last fractions correspond to the BIONs release from the system.

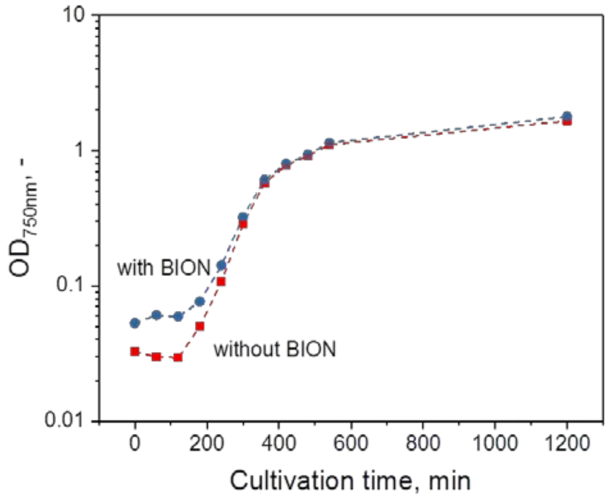

Figure S10: In-situ cultivation experiments with 85 mL *Saccharomyces cerevisiae* in YPD-Medium (30°C, 220 rpm shaker)**.**
